# Supplementary material for: Prognostic Significance of MRE11 Overexpression in Colorectal Cancer Patients
Source: Cancers (Basel). 2023 Apr 24;15(9):2438. doi: 10.3390/cancers15092438 (PMC10177562; doi:10.3390/cancers15092438)
Supplement: Supplementary file 1 [file cancers-15-02438-s001.zip › cancers-2317478-supplementary.pdf]

Supplementary Materials: Prognostic significance of MRE11 overexpression in colorectal cancer patients

Vincent Ho [1,2,¶,*], Liping Chung [1,2,¶], Kate Wilkinson [2,3], Vivienne Lea[1,3], Stephanie H Lim [2,4,5], Askar Abubakar [1,2], Weng Ng [5], Mark Lee [6], Tara L Roberts [1,2,7], Wei Chua [1,5,8] and Cheok Soon Lee [1,2,3,7,9]

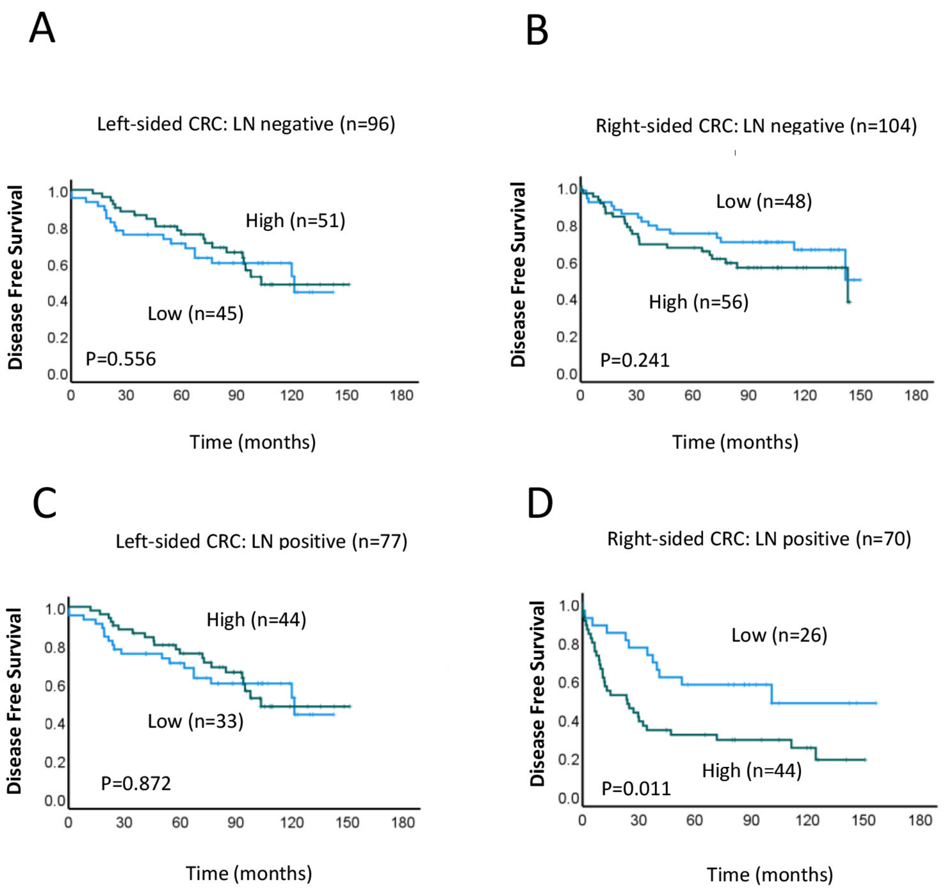

Supplementary Figure S1. Association between MRE11 expression and survival, based on location of the primary colorectal cancer (CRC) in relation to lymph node (LN) involvement. (A–D) Kaplan–Meier analysis of disease-free survival in patients with high (green line) or low (blue line) MRE11 expression in left-sided (A, LN-negative; C, LN-positive) and right-sided (B, LN-negative; D, LN-positive) CRC.
